# The association between embedded catheter implantation and hospitalization costs for peritoneal dialysis initiation: a retrospective cohort study

**DOI:** 10.1007/s10157-023-02416-z

**Published:** 2023-11-14

**Authors:** Maki Shinzawa, Ayumi Matsumoto, Harumi Kitamura, Yusuke Sakaguchi, Atsushi Takahashi, Isao Matsui, Masayuki Mizui, Ryohei Yamamoto, Yoshitaka Isaka

**Affiliations:** 1 Department of Nephrology, Osaka University Graduate School of Medicine, D-11 2-2 Yamadaoka, Suita, Osaka 565-0871 Japan; 2 Health and Counseling Center, Osaka University, Toyonaka, Japan; 3 Department of Inter-Organ Communication Research in Kidney Disease, Osaka University Graduate School of Medicine, Suita, Japan; 4 Laboratory of Behavioral Health Promotion, Department of Health Promotion Medicine , Osaka University Graduate School of Medicine, Toyonaka, Japan

**Keywords:** Peritoneal dialysis, Initiation, Stepwise initiation of PD using Moncrief and Popovich’s technique (SMAP), Embedding catheter, Hospitalization cost, Length of hospitalization

## Abstract

**Background:**

Compared with the conventional peritoneal dialysis (PD) catheter insertion, embedding PD catheter implantation is one of the procedures for planned PD initiation. However, facilities where embedded PD catheter implantation is available are limited, and the impact of embedded PD catheter implantation on hospitalization cost and length of hospitalization is unknown.

**Methods:**

This retrospective single-center cohort study included 132 patients with PD initiation between 2005 and 2020. The patients were divided into two groups: 64 patients in the embedding group and 68 patients in the conventional insertion group. We created a multivariable generalized linear model (GLM) with the gamma family and log-link function to evaluate the association among catheter embedding, the duration and medical costs of hospitalization for PD initiation. We also evaluated the effect modification between age and catheter embedding.

**Results:**

Catheter embedding (*β* coefficient − 0.13 [95% confidence interval − 0.21, − 0.05]) and age (per 10 years 0.08 [0.03, 0.14]) were significantly associated with hospitalization costs. Catheter embedding (− 0.21 [− 0.32, − 0.10]) and age (0.11 [0.03, 0.19]) were also identified as factors significantly associated with length of hospitalization. The difference between the embedding group and the conventional insertion group in hospitalization costs for PD initiation (*P* for interaction = 0.060) and the length of hospitalization (*P* for interaction = 0.027) was larger in young-to-middle-aged patients than in elderly patients.

**Conclusions:**

Catheter embedding was associated with lower hospitalization cost and shorter length of hospitalization for PD initiation than conventional PD catheter insertion, especially in young-to-middle-aged patients.

## Introduction

With the increasing number of patients with end-stage kidney disease (ESKD) undergoing dialysis, the cost of medical care for patients with dialysis is increasing worldwide, creating an enormous economic burden [1–3]. According to the United States Renal Data System (USRDS) 2021 Annual Data Report, healthcare expenditures for patients with hemodialysis (HD) and peritoneal dialysis (PD) increased: total expenditures for Medicare fee-for-service beneficiaries with ESRD increased from $28.0 billion in 2009 to $37.3 billion in 2019 [4]. Compared with patients with PD, healthcare expenditure per person was higher in patients with HD [5]. In addition, medical expenditure for unplanned urgent HD initiation without planned creation of vascular access is higher than that for planned HD initiation with planned creation of vascular access [6–8], indicating the importance of planned HD initiation in reducing healthcare expenditure.

Catheter embedding is one of the procedures for planned PD initiation [9, 10], sometimes called SMAP: Stepwise initiation of PD using Moncrief and Popovich’s technique [10–13]. Catheter embedding comprises two steps: embedded catheter implantation and catheter externalization with exit-site catheter fixation. Compared with conventional PD catheter insertion, catheter embedding has several advantages or maintaining a long break-in period. First, although catheter maintenance is required after conventional PD catheter insertion, catheter embedding does not require catheter maintenance care during the break-in period [9, 10]. The break-in period is defined as the time interval between PD catheter insertion and PD initiation. Second, patients with catheter embedded are more likely to be committed to PD and comprehensive patient education during the break-in period [9, 14]. Third, because of the extended healing time for embedded catheter implantation, catheter embedding can suppress tunnel infection and catheter infection-related peritonitis after PD initiation [12]. However, the effect of catheter embedding on hospitalization costs for PD initiation has not been assessed.

The aim of the present study was to evaluate the effect of catheter embedding on the hospitalization costs for PD initiation in 132 patients compared with the conventional PD catheter insertion. We expect our results to provide meaningful insights into treatment strategies for PD initiation using catheter embedding.

## Methods

### Setting and participants

Eligible for the present study were 142 patients who initiated PD at the Department of Nephrology, Osaka University Hospital, Suita City, Japan, from April 2005 to March 2020. After excluding 10 patients (7.0%) who underwent PD catheter insertion at other hospitals, we included 132 patients in the present study, including 64 patients in the embedding group and 68 patients in the conventional insertion group.

The study protocol was approved by the Osaka University Hospital (No. 21266). Because of the observational nature of the study, our hospital used an opt-out approach to provide informed consent, according to the Japanese Ethical Guidelines for Medical and Health Research Involving Human Subjects.

### Measurements

The baseline variables included age, sex, body mass index, systolic and diastolic blood pressure, serum creatinine, estimated Glomerular Filtration Rate (eGFR), history of diabetes mellitus, use of anti-hypertensives, and lipid-lowering drugs in the embedding group and the conventional insertion group at the date of PD initiation. The date for the conventional insertion group was set at the date of performing a catheter insertion, and the date for the embedding group was set at the date of performing a catheter externalization with an exit-site catheter fixation. The formula for calculating eGFR was as follows: eGFR (mL/min/1.73 m^2^) = 194 × serum creatine (mg/dL)^−1.094^ × age (years)^−0.287^ × 0.739 (if female) [15]. All catheter insertions and embedding surgeries used laparoscopy to insert the tip of the catheter into the pouch of Douglas. The baseline variables additionally included PD-related information: early referral, PD dialysate brand, use of a device for PD catheter connection, use of an automated PD system (APD), the incidence of HD before PD initiation, the length of days from PD catheter insertion to PD initiation, and the primary failure rate of PD catheters (Table 1). The KDOQI guidelines [16] refined that the definition of early referral was at least 3 months, which was the absolute minimum amount of time for assessment, education, preparation for renal replacement therapy, and creation of access. Accordingly, early referral was defined as less than 90 days from first visit at our department to a catheter insertion or externalization. The use of a device for PD catheter connection was defined as the use of a connection assistant device: Terumo Sterile Connecting Device^®^ (TSCD^®^), TSUNAGU^®^, and U. V. Flash AUTO^®^. The primary failure rate of PD catheters was defined as the number of procedures or surgeries, or both, required to solve catheter problems during hospitalization for PD initiation.

**Table 1:** Baseline characteristics at PD initiation

	Embedding *N* = 64	Conventional insertion *N* = 68	*P* value
Age, years	63 (52–68)	54 (47–65)	0.046
Male, *n* (%)	42 (65.6)	38 (55.9)	0.252
Body mass index, Kg/m^2^	22.9 ± 3.9	22.6 ± 3.7	0.670
Systolic blood pressure, mmHg	135 ± 19	136 ± 23	0.841
Diastolic blood pressure, mmHg	75 ± 12	77 ± 12	0.336
Serum creatinine, mg/dL	8.9 (8.1–10.1)	9.5 (8.1–11.0)	0.149
eGFR, mL/min/1.73 m^2^	5.0 ± 1.5	4.7 ± 1.2	0.211
History of diabetes mellitus, *n* (%)	20 (31.3)	20 (29.4)	0.818
Use of anti-hypertensives, *n* (%)	62 (96.9)	66 (97.1)	0.951
Use of lipid-lowering drugs, *n* (%)	26 (40.6)	29 (42.6)	0.814
PD-related information			
Early referral, *n* (%)	2 (3.1)	17 (25.0)	< 0.001
PD dialysate brand			0.117
Baxter, *n* (%)	51 (76.7)	46 (67.6)	
Terumo, *n* (%)	13 (20.3)	22 (32.4)	
Use of a device for PD catheter connection, *n* (%)	27 (42.2)	29 (42.6)	0.957
Use of APD, *n* (%)	28 (43.8)	29 (42.6)	0.898
HD before PD initiation, *n* (%)	1 (1.6)	11 (16.2)	0.004
Length of days after PD catheter insertion, days	128 (74–219)		
Primary failure rate, *n* (%)	3 (4.7)	0 (0)	0.071

Data are presented as mean ± standard deviation or median (interquartile range) for continuous measures, and *n* (%) for categorical measures

*APD* automated peritoneal dialysis system, *eGFR* estimated glomerular filtration rate, *HD* hemodialysis, *PD* peritoneal dialysis

**Fig. 1:**
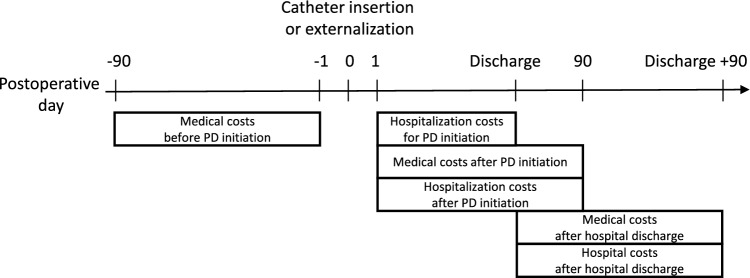
The definition to calculate the hospitalization and medical costs before and after PD initiation

The main outcome of the present study was the hospitalization cost for PD initiation, which were calculated from the first postoperative day after a catheter insertion or externalization to the hospital discharge day for PD initiation, namely the hospitalization cost did not include the cost of catheter insertion or externalization (Fig. 1). Clinical pathways for PD initiation were not used in our department, and therefore, each physician clinically determined the hospital discharge date for based on the patient's condition. The 90-day period of medical costs included outpatient and inpatient costs before or after PD initiation. To assess the PD initiation period, we evaluated hospitalization and medical costs, hospital readmission, and length of hospitalization during the 90-day period after PD initiation. Hospital readmission was defined as admission to our hospital, regardless of whether the patient was admitted to other departments or the department of nephrology. In addition, we evaluated the outcomes at 90 days after hospital discharge for PD initiation. We calculated all costs at 130 yen to the dollar.

### Statistical analysis

We compared baseline characteristics between the embedding group and the conventional insertion group using Student’s *t* test or the Wilcoxon rank-sum test for continuous variables and the chi-squared test for categorical variables, as appropriate.

To assess the association of catheter embedding with the duration and medical costs of hospitalization for PD initiation, we created a univariable and multivariable generalized linear model (GLM) with the gamma family and log-link function with clustering by every 2 years of PD initiation for a revision of the medical payment system in Japan. Multivariable GLMs were adjusted for age (years), sex, body mass index (kg/m^2^), mean arterial pressure (mmHg), eGFR (mL/min/1.73 m^2^), history of diabetes mellitus, use of antihypertensive agents, use of lipid-lowering drugs, PD dialysate brand, use of a device for PD catheter connection, use of APD, and the incidence of HD before PD initiation. We calculated the variance inflation factor (VIF), which was less than 10, indicating no multicollinearity of variables in all models in this study [17]. To examine the goodness of fit (GoF) of each GLM, we used of Pregibon’s link test using the STATA command “linktest” [18]. To estimate the 95% confidence interval (95% CI), we used bootstrapping method with 1000 repetitions with replacement. To exclude the effect of early referral on the outcomes, the association of catheter embedding and age with outcomes was assessed in patients without early referral.

The effect modification between catheter embedding and age was assessed by incorporating the interaction term “age × catheter embedding” into the multivariable GLM. To estimate the prediction of the hospitalization costs and length of hospitalization for PD initiation and calculate the marginal effects of catheter embedding and age on the confounders, we used STATA commands: “margins” and “marginsplot” [19]. To assess the potential mediating effects of the length of hospitalization for PD initiation on the relationship among catheter embedding, age, and hospitalization costs for PD initiation, we evaluated the presence of attenuation in the effect of catheter embedding and age on the hospitalization costs for PD initiation after adjustment for the potential mediator, the length of hospitalization for PD initiation, and confounders.

Continuous variables are expressed as mean ± standard deviation or median (interquartile range) appropriately, while categorical variables are expressed as numbers and proportions. All *P* values were two-tailed, and statistical significance was set at *P* < 0.05. For the interaction *P* value, statistical significance was set at 0.1 level [20]. All statistical analyses were conducted using STATA version 16.1 (STATA Corp., College Station, TX, USA).

## Results

The baseline characteristics of the embedding (n = 64) and conventional insertion (*n* = 68) groups are presented in Table 1. The embedding group was significantly older than the conventional insertion group (median 63 [interquartile range 52–68] vs. 54 [47–65] years, *P* = 0.046). Other baseline characteristics were comparable between the two groups. Regarding PD-related information, the embedding group had a significantly lower prevalence of early referral (2 [3.1%] vs. 17 [25.0%], *P* < 0.001) and hemodialysis before PD initiation (1 [1.6%] vs. 11 [16.2%], *P* = 0.004). In the embedding group, the length of days from PD catheter insertion to PD initiation was 128 (74–219) days. Three patients in the embedding group, including 2 patients with fibrin clots and 1 patient with omental wrapping, had primary PD catheter failure in 157th, 480th, and 630th days after PD catheter embedding, respectively. Whereas no patient in the conventional insertion group experienced primary PD catheter failure.

The hospitalization costs from the 1st to 5th or 7th postoperative day in the embedding group were significantly higher than in the conventional insertion group (1st to 5th postoperative day: $1,767 [1,703–1,946] vs. $1,639 [1,504–1,765], *P* < 0.001; 1st to 7th postoperative day: $2,474 [2,299–2,704] vs. $2,285 [2,135–2,472], *P* = 0.001). However, the embedding group had lower hospitalization costs for PD initiation ($8,171 [6,165–11,285] vs. $9,849 [8,542–11,429], *P* = 0.003) and a shorter length of hospitalization for PD initiation (20 [16–29] days vs. 30 [23–34] days, *P* < 0.001) than the conventional insertion group (Table 2). At the 90-day period before PD initiation, medical costs were significantly lower in the embedding group ($2,815 [2,299–7,302] vs. $4,562 [4,049–6,256], *P* < 0.001); however, medical costs after PD initiation and hospital discharge were not statistically significant between the embedding group and the conventional insertion group. Similarly, hospital readmissions to our department and all other departments were not significantly different between the two groups.

**Table 2:** The number of days for hospitalization and medical costs

	Embedding *N* = 64	Conventional insertion *N* = 68	*P* value
90-day period before PD initiation			
Medical costs before PD initiation, $	2,815 (2,299–7,302)	4,562 (4,049–6,256)	< 0.001
Hospitalization period for PD initiation			
Hospitalization costs during PD initiation, $	8,171 (6,165–11,285)	9,849 (8,542–11,429)	0.003
Hospitalization costs from 1st to 5th postoperative day, $	1,767 (1,703–1,946)	1,639 (1,504–1,765)	< 0.001
Hospitalization costs from 1st to 7th postoperative day, $	2,474 (2,299–2,704)	2,285 (2,135–2,472)	0.001
Length of hospitalization for PD initiation, days	20 (16–29)	30 (23, 34)	< 0.001
90-day period after PD initiation			
Medical costs after PD initiation, $	10,988 (8,169–13,779)	11,706 (10,216–13,374)	0.161
Hospital readmission to the department of nephrology, *n* (%)	9 (14.1)	6 (8.8)	0.343
Hospital readmission to all department, *n* (%)	13 (20.3)	6 (8.8)	0.060
Length of hospitalization in the department of nephrology, days	21 (16–32)	31 (23–36)	< 0.001
Length of hospitalization in all department, days	21 (17–34)	31 (23–36)	0.002
Hospitalization costs after PD initiation, $	8,726 (6,646–12,522)	10,104 (8,635–11,953)	0.095
90-day period after hospital discharge for PD initiation			
Medical costs after hospital discharge, $	2,555 (1,952–3,864)	2,405 (1,945–3,119)	0.536
Hospital readmission to the department of nephrology, *n* (%)	11 (17.2)	9 (13.2)	0.527
Hospital readmission to all department, *n* (%)	15 (23.4)	10 (14.7)	0.201
Length of hospitalization in the department of nephrology, days	0 (0–0)	0 (0–0)	0.552
Length of hospitalization in all department, days	0 (0–0)	0 (0–0)	0.229
Hospitalization costs after hospital discharge, $	5,077 (2,116–10,236)	7,215 (3,263–10,425)	0.598

*PD* peritoneal dialysis

To identify the contributors to hospitalization costs for PD initiation, we employed multivariable GLMs. Catheter embedding (− 0.13 [95% CI − 0.21 to − 0.05], *P* = 0.002) and age (per 10 years 0.08 [0.03 to 0.14], *P* = 0.002) were significantly associated with hospitalization costs for PD initiation (Table 3a, Model 2). Catheter embedding (− 0.21 [− 0.32 to − 0.10], *P* < 0.001) and age (per 10 years 0.11 [0.03 to 0.19], *P* = 0.005) were also identified as factors significantly associated with the length of hospitalization for PD initiation (Table 3b, Model 2). Multivariable GLMs using bootstrap methods verified the significant association of catheter embedding and age with hospitalization costs and length of hospitalization for PD initiation (Model 3 in Table 3a, b). After excluding 19 patients with early referral, catheter embedding and age were similarly associated with the outcomes (the hospitalization cost: catheter embedding, − 0.17 [− 0.28 to − 0.06], *P* = 0.002 and age, per 10 years 0.10 [0.05–0.16], *P* < 0.001; the length of hospitalization: catheter embedding, − 0.24 [− 0.35 to − 0.13], *P* < 0.001 and age, per 10 years 0.11 [0.03–0.20], *P* = 0.007).

**Table 3:** The association with hospitalization costs and the length of hospitalization for PD initiation

	Model 1 *β* (95% CI)	*P* value	Model 2 *β* (95% CI)	*P* value	Model 3 *β* (95% CI)	*P* value
(a) The association of hospitalization costs for PD initiation						
Catheter embedding	− 0.11 (− 0.22 to 0.01)	0.069	− 0.13 (− 0.21 to − 0.05)	0.002	− 0.13 (− 0.21 to − 0.05)	0.002
Age, 10 years	0.07 (0.02 to 0.13)	0.011	0.08 (0.03 to 0.14)	0.002	0.08 (0.03 to 0.13)	0.001
Male	0.03 (− 0.03 to 0.10)	0.326	0.08 (− 0.03 to 0.20)	0.148	0.08 (− 0.04 to 0.21)	0.195
Body mass index, Kg/m^2^	0.00 (− 0.01 to 0.01)	0.971	0 (− 0.01 to 0.01)	0.590	0.00 (− 0.01 to 0.01)	0.672
Mean arterial pressure, 10 mmHg	0.04 (0.00 to 0.08)	0.075	0.02 (− 0.01 to 0.04)	0.279	0.02 (− 0.02 to 0.05)	0.354
eGFR, mL/min/1.73 m^2^	− 0.04 (− 0.08 to − 0.01)	0.006	− 0.04 (− 0.06 to − 0.02)	< 0.001	− 0.04 (− 0.07 to − 0.02)	< 0.001
History of diabetes mellitus	0.00 (− 0.11 to 0.11)	0.978	− 0.02 (− 0.15 to 0.10)	0.719	− 0.02 (− 0.16 to 0.11)	0.739
Use of anti-hypertensives	− 0.57 (− 1.02 to − 0.12)	0.013	− 0.43 (− 0.79 to − 0.07)	0.019	− 0.43 (− 0.83 to − 0.03)	0.035
Use of lipid-lowering drugs	0.02 (− 0.07 to 0.11)	0.659	0.00 (− 0.08 to 0.08)	0.956	0.00 (− 0.09 to 0.10)	0.963
Terumo	0.09 (− 0.04 to 0.23)	0.183	0.11 (0.02 to 0.21)	0.020	0.11 (− 0.01 to 0.24)	0.072
Use of a device for PD catheter connection	0.21 (0.13 to 0.30)	< 0.001	0.12 (0.02 to 0.21)	0.018	0.12 (0.02 to 0.21)	0.018
Use of APD	0.11 (0.01 to 0.21)	0.031	0.13 (0.03 to 0.22)	0.009	0.13 (0.03 to 0.22)	0.010
HD before PD initiation	0.09 (− 0.01 to 0.20)	0.085	0.13 (0.00 to 0.25)	0.052	0.13 (− 0.01 to 0.27)	0.075
(b) The association of length of hospitalization for PD initiation						
Catheter embedding	− 0.18 (− 0.30 to − 0.05)	0.005	− 0.21 (− 0.32 to − 0.10)	< 0.001	− 0.21 (− 0.31 to − 0.11)	< 0.001
Age, 10 years	0.10 (0.03 to 0.17)	0.008	0.11 (0.03 to 0.19)	0.005	0.11 (0.03 to 0.18)	0.004
Male	− 0.05 (− 0.17 to 0.06)	0.359	− 0.03 (− 0.18 to 0.12)	0.683	− 0.03 (− 0.18 to 0.12)	0.691
Body mass index, Kg/m^2^	0.00 (− 0.02 to 0.02)	0.980	0.01 (− 0.01 to 0.02)	0.295	0.01 (− 0.01 to 0.03)	0.377
Mean arterial pressure, 10 mmHg	0.05 (0.01 to 0.09)	0.016	0.03 (0.00 to 0.07)	0.081	0.03 (− 0.01 to 0.08)	0.150
eGFR, mL/min/1.73 m^2^	− 0.03 (− 0.06 to 0.00)	0.055	− 0.01 (− 0.05 to 0.03)	0.549	− 0.01 (− 0.05 to 0.03)	0.590
History of diabetes mellitus	0.04 (− 0.11 to 0.18)	0.619	− 0.01 (− 0.15 to 0.14)	0.945	− 0.01 (− 0.17 to 0.16)	0.950
Use of anti-hypertensives	− 0.46 (− 0.97 to 0.05)	0.078	− 0.38 (− 0.89 to 0.13)	0.141	− 0.38 (− 0.93 to 0.17)	0.174
Use of lipid-lowering drugs	0.05 (− 0.06 to 0.16)	0.387	0.00 (− 0.11 to 0.10)	0.958	0.00 (− 0.12 to 0.12)	0.964
Terumo	0.12 (− 0.02 to 0.27)	0.092	0.09 (− 0.05 to 0.23)	0.209	0.09 (− 0.10 to 0.28)	0.358
Use of a device for PD catheter connection	0.22 (0.14 to 0.31)	< 0.001	0.11 (0.01 to 0.22)	0.031	0.11 (0.00 to 0.22)	0.043
Use of APD	− 0.01 (− 0.13 to 0.10)	0.839	0.02 (− 0.13 to 0.17)	0.780	0.02 (− 0.13 to 0.17)	0.781
HD before PD initiation	0.16 (− 0.01 to 0.33)	0.063	0.19 (0.07 to 0.32)	0.002	0.19 (0.05 to 0.34)	0.010

Model 1: univariable model; Model 2: multivariable model; Model 3 multivariable model using bootstrap methods

APD: Automated peritoneal dialysis system; CI: confidence interval; eGFR: estimated Glomerular Filtration Rate; HD: hemodialysis; PD: peritoneal dialysis; β: β coefficient

**Fig. 2:**
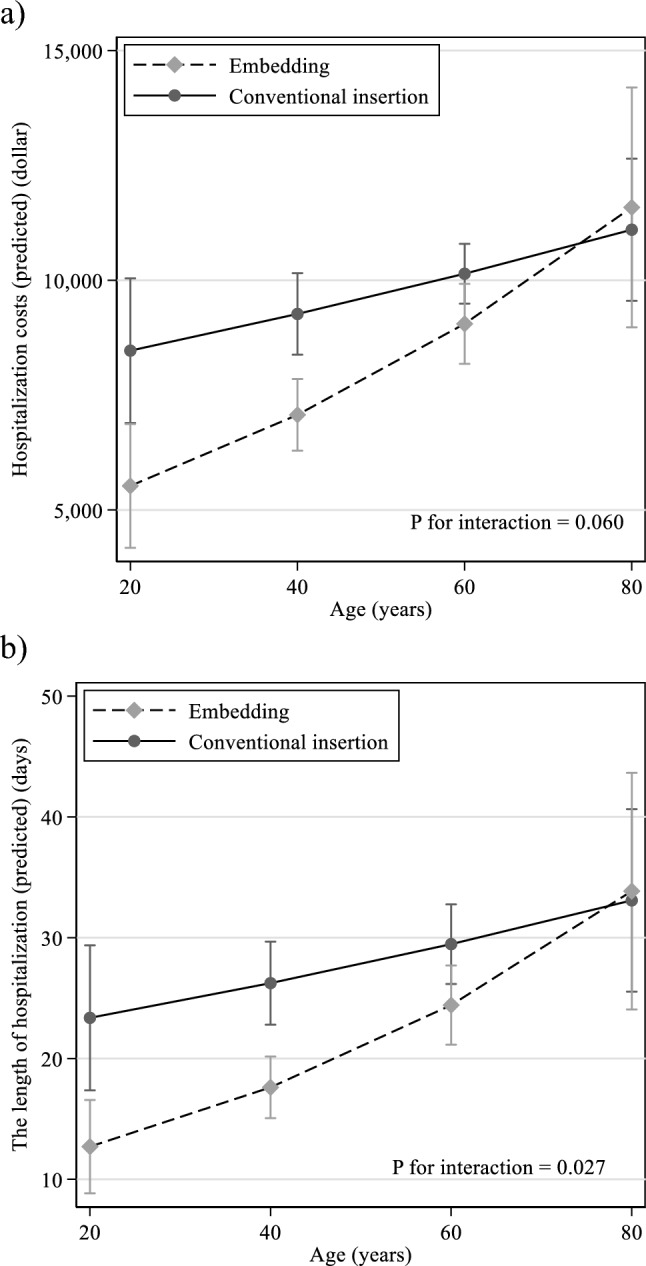
Predictive margins for the relationship between age and the hospitalization costs during PD initiation (**a**) and between age and the length of hospitalization for PD initiation (**b**)

Predicted average hospitalization costs for PD initiation are listed in Table 4, $8,825 (8,059–9,590) in the embedding group and $10,021 (9,423–10,620) in the conventional insertion group. As age increased, hospitalization costs for PD initiation also increased in both groups. Interestingly, the difference between the embedding and conventional insertion groups in hospitalization costs for PD initiation was larger in young to middle-aged patients than in elderly patients (*P* for interaction = 0.060 in Fig. 2a). Regarding the length of hospitalization for PD initiation, the predicted average length of hospitalization for PD initiation was 24 (21–27) days in the embedding group and 29 (26–32) days in the conventional insertion group. A similar effect modification of the length of hospitalization for PD initiation was observed between the embedding and conventional insertion groups (*P* for interaction = 0.027 in Fig. 2b)

.

**Table 4:** Predicted the hospitalization costs and the length of hospitalization for PD initiation

Age	Embedding *N* = 64 Margin (95% CI)	Conventional insertion *N* = 68 Margin (95% CI)
(a) Predicted the hospitalization costs for PD initiation ($)		
All	8,825 (8,059–9,590)	10,021 (9,423–10,620)
20 years	5,524 (4,176–6,873)	8,468 (6,894–10,042)
40 years	7,071 (6,291–7,851)	9,267 (8,381–10,154)
60 years	9,051 (8,182–9,921)	10,142 (9,492–10,793)
80 years	11,586 (8,976–14,197)	11,100 (9,552–12,647)
(b) Predicted the length of hospitalization for PD initiation (days)		
All	24 (21–27)	29 (26–32)
20 years	13 (9–17)	23 (17–29)
40 years	18 (15–20)	26 (23–30)
60 years	24 (21–28)	30 (26–33)
80 years	34 (24–44)	33 (26–41)

*CI* confidence interval

To identify the association between the length of hospitalization and hospitalization cost, we evaluated the potential mediating effects of the length of hospitalization for PD initiation on the association of catheter embedding and age with hospitalization costs. In Table 3a, b, catheter embedding and age were independently associated with hospitalization costs and length of hospitalization for PD initiation. We then performed a multivariable GLM using bootstrap methods, including the length of hospitalization for PD initiation and the same confounders shown in Table 3a, b. After including the length of hospitalization for PD initiation in the multivariable GLM using bootstrap methods, the length of hospitalization for PD initiation was significantly associated with hospitalization cost for PD initiation (0.02 [0.02–0.03], *P* < 0.001), whereas the association between catheter embedding and age was attenuated; catheter embedding (− 0.03 [− 0.09 to 0.03)], *P* = 0.280) and age (0.01 [− 0.02 to 0.03], *P* = 0.528), suggesting that the length of hospitalization for PD initiation was a potential mediator of hospitalization costs for catheter embedding and age regardless of a catheter insertion or externalization.

## Discussion

During the PD initiation period in the embedding group, the hospitalization costs were significantly lower, and the length of hospitalization was significantly shorter than that in the conventional insertion group. Catheter embedding and age were identified as contributors to hospitalization costs and the length of hospitalization for PD initiation. The hospitalization costs and length of hospitalization for PD initiation increased with age in both the embedding and the conventional insertion groups. Especially in young-to-middle-aged patients with PD, the embedding group had lower hospitalization costs for PD initiation than the conventional insertion group. Whereas the costs were comparable between the two groups in elderly patients with PD. The same effect modification was observed in the association of catheter embedding and age with the length of hospitalization for PD initiation. In addition to performing the multivariable GLM using bootstrap methods, including the length of hospitalization for PD initiation, catheter embedding, age, and confounders, the length of hospitalization for PD initiation was a potential mediator of the hospitalization costs for catheter embedding and age.

The Japanese Society for PD (JSPD) guideline [10] stated that planned PD initiation is characterized by a shorter duration of hospitalization and lower hospitalization cost than unplanned urgent PD initiation. However, a few studies have reported an association of planned or unplanned urgent PD initiation with the length of hospitalization or hospitalization cost for PD initiation, or both. A retrospective cohort study including 310 patients with PD in a single Chinese hospital was aimed at investigating the feasibility of shorter break-in periods following PD catheter insertion from 2003 to 2007 [21]. The authors divided patients with PD initiation into two groups: 226 patients with PD initiation in the early initiation group, which was unplanned urgent PD initiation and defined as break-in period ≤ 14 days, and 84 patients in the late initiation group, which was planned PD initiation and defined as brake-in period > 14 days. In their study, the early initiation (short break-in period) group was associated with long hospitalization for PD initiation; 11.8 ± 10.2 days for the early initiation group and 7.5 ± 6.2 days for the late initiation group (*P* < 0.001). The present study divided patients with PD initiation into the embedded group, which is one of the procedures for planned PD initiation, and the conventional insertion group, whose break-in period was shorter than that of the embedding group. The length of hospitalization for planned PD initiation, including that for the embedded catheter, was shorter than that for unplanned urgent PD initiation. Moreover, a few studies have reported the medical costs associated with PD initiation. The present study reported the medical cost of PD initiation between the embedding group and the conventional insertion group. In addition, despite higher hospitalization costs from the 1st to 5th or 7th postoperative day in the embedding group, the hospitalization costs for PD initiation were lower in the embedding group than in the conventional insertion group.

PD patients are particularly required to become skilled at PD exchange and patient education, mainly on the physiological mechanism of PD, training for exchange procedures, and troubleshooting [14, 22]. For elderly patients, obtaining some medical techniques and learning knowledge of their disease is harder than young to middle-aged patients [23]. A Spanish cohort study including 135 patients mainly with planned PD initiation at a university hospital reported that the median training sessions for PD were 10 (interquartile range 8–13) and the training duration was 19 days (interquartile range 14–28) [24]. In their study, 31 patients (23%) needed prolonged training sessions, defined as more than 13 training sessions. Compared with the usual training sessions group, the prolonged training sessions group was significantly older (64.9 ± 15.1 vs. 51.6 ± 14.6, *P* < 0.05), consistent with the findings of the present study. In the present study, age was also affected by the length of hospitalization for PD initiation. The results of their study strongly suggest that elderly patients required longer training, reading to the longer hospitalization and higher medical costs. In addition to age, catheter embedding affected the hospitalization costs and the length of hospitalization for PD initiation. Moreover, we revealed the effect modification between catheter embedding and age on hospitalization costs and the length of hospitalization for PD initiation. The embedding group had lower hospitalization costs and shorter lengths of hospitalization for PD initiation in young-to-middle-aged patients with PD than the conventional insertion group, while in elderly patients, the length of hospitalization and the hospitalization cost of the embedding group were comparable to those of the conventional insertion group. The results would indicate that more PD training is required in elderly patients than in the young-to-middle-aged patients. This attenuated the effect of a shorter length of hospitalization in the elderly embedding group; the advantage of catheter embedding was diminished.

The present study had several limitations. First, patient education on PD initiation, which was presented by the ISPD guideline [14], is one of the most important factors for the length of hospitalization and is affected by age, comorbidity, cognitive function, and patients’ socio-economic status (i.e., educational level, occupation, and income data) [25]. However, our data were based on medical claims data and laboratory data at a single center, and we had no information about the cognitive functions and socio-economic status of the patients. Second, the Japanese universal health insurance coverage system covered all citizens through public medical insurance, and the healthcare insurance system differed in each country. Third, we did not evaluate the association between PD initiation by catheter embedding and long-term PD outcomes (duration of PD, peritonitis, and residual renal function). Fourth, the present study had no information on the format of the PD training sessions. Further studies are needed to assess the impact of patient education on the length of hospitalization and hospitalization costs for PD initiation in young and elderly patients.

In conclusion, the present retrospective cohort study identified that catheter embedding and age affected the hospitalization cost and length of hospitalization for PD initiation in 132 patients compared with the conventional PD catheter insertion. The results suggest that catheter embedding for PD initiation in young-to-middle-aged patients have more advantages in terms of hospitalization cost and length of hospitalization for PD initiation.

## Data Availability

The data that support the findings of this study are available on request from the corresponding author, MS. The data are not publicly available due to the privacy of pariticipants or ethical restrictions.
